# Venous obstruction of thyroid malignancy origin: the Antoine Lacassagne Institute experience

**DOI:** 10.1186/1477-7819-7-40

**Published:** 2009-04-17

**Authors:** Pierre-Yves René Marcy, Juliet Thariat, Alex Bozec, Gilles Poissonnet, Danielle Benisvy, Olivier Dassonville

**Affiliations:** 1 Head § Neck Oncology Imaging Department, Antoine Lacassagne Cancer Research Institute, 33 Avenue Valombrose, 06189, Nice, cedex1, France; 2 Radiation Therapy Department, Antoine Lacassagne Cancer Research Institute, 33 Avenue Valombrose, 06189, Nice, cedex1, France; 3 Head § Neck Oncology Surgery Department, Antoine Lacassagne Cancer Research Institute, 33 Avenue Valombrose, 06189, Nice, cedex1, France; 4 Nuclear Medicine Department, Antoine Lacassagne Cancer Research Institute, 33 Avenue Valombrose, 06189, Nice, cedex1, France

## Abstract

**Background and aims:**

To show the benefits of Ultrasonography in the diagnosis of great vein involvement in the neck and mediastinum in thyroid malignancies (primary or secondary) in our experience and to report patient outcomes.

**Methods:**

Clinical data were collected from the thyroid unit database of the Antoine Lacassagne Institute.

**Results:**

Of 1171 patients with thyroid cancer treated at our institution over the last 18 years, we retrospectively identified nine patients (0.8%), three women and six men, aged 34–81 years (median age: 70 years) presenting with malignant thyroid tumor of median diameter 45 mm (range: 23–87) having venous obstruction of thyroid malignancy origin. Two patients underwent multimodal therapy. All other patients underwent external beam radiation therapy alone ± chemotherapy or palliative care. Ultrasound (US) provided particularly useful information on venous involvement characteristics. Median survival was 7 months and median progression-free survival was 6 months. Survival in our series was worse than that of previously reported series despite diagnosis of vein involvement at an early stage in 2/3 cases using US.

**Conclusion:**

Despite small numbers of patients, it seems that aggressive treatment modalities including surgery are required to improve survival. In our experience, US was a useful non-invasive method to describe tumor extensions to great veins of the neck (invasion versus compression, tumor thrombus versus blood clot) and should be recommended to depict early venous invasion in cases of suspected thyroid malignancy.

## Background

Superior vena cava (SVC) obstruction is associated with lung cancer, malignant lymphoma and mediastinal metastases. In less than 1% of the cases, SVC syndrome (SVCS) is due to massive invasion into the great veins or compression of the SVC by a thyroid cancer [1]. Only 29 cases have been reported in the literature so far. We hereby report on the Antoine Lacassagne Institute's experience and provide additional data on neck Ultrasonography (US) and patient outcomes in our series, in which most of patients could not undergo curative treatment. Clinical features, tumor size, histological types, and outcomes to therapy are presented.

## Materials and methods

From 1991 to 2008, clinical and radiological data were collected from the thyroid unit database of the Antoine Lacassagne Institute. All patients had vein assessment on systematic Doppler US of the neck at initial diagnosis work-up and follow-up, and cross-sectional imaging scans to assess tumor extensions.

## Results

**Figure 1:**
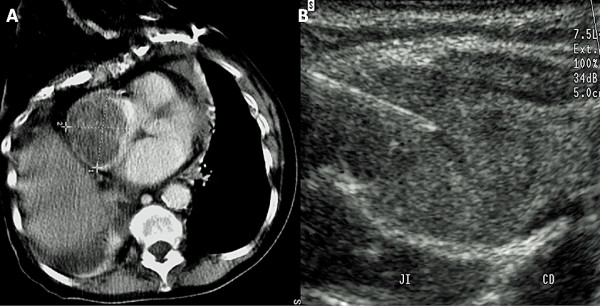
**Case 2 – (A) Thorax axial single slice-CT in an anaplastic thyroid carcinoma patient invading the SVC and right atrium**. CT scan showed giant tumor mass extending into the right atrium (35 mm). Tumor thrombus originated in the neck. **(B)** Neck tumor biopsied under real-time US guidance. Carotid artery (CD) and internal jugular vein (JI) were compressed at the upper border of the tumor. Subclavian vein Doppler assessment depicted damping waveforms in both SCV (right and left), thus indicating a high probability of SVC obstruction [5].

**Figure 2:**
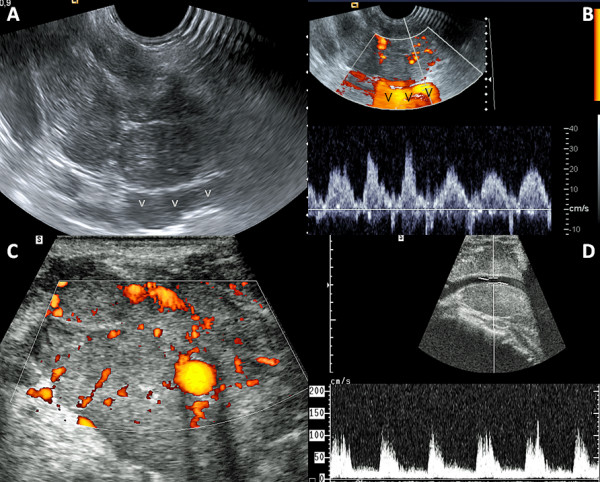
**Case 1 – (A, B) Doppler US scan of poorly differentiated extensive papillary thyroid carcinoma at the level of the supra-sternal notch**. **(A) **US scan Using a craniopodal orientation of the "endocavitary probe", the shape of this "specific probe" used here allows for visualization of compressed left inominate vein (v) by the tumor and malignant nodes at level VII. **(B) **(same patient, same area of interest). Color Doppler assessment of left inominate vein shows persistent respiratory phasicity and cardiac rythmicity. This indicates patency of the inominate vein and SVC [5]. **(C) **Axial Color Doppler in the left neck in a T4b thyroid cancer patient shows left common carotid artery (LCA) encasement by the aggressive tumor. A 360° encasement was a local contraindication to local surgery at that time, at our institution. The concept of "shave resection" was established a few years later. **(D) **Longitudinal Doppler US scan shows a typical waveform identifying tumoral stenosis of LCA. Left IJV was compressed by tumor. Right SCV Doppler assessment showed normal Doppler waveforms. Thus, central venous compression was localized to the left side of the neck and upper mediastinum without thrombosis of the SVC [5].

**Figure 3:**
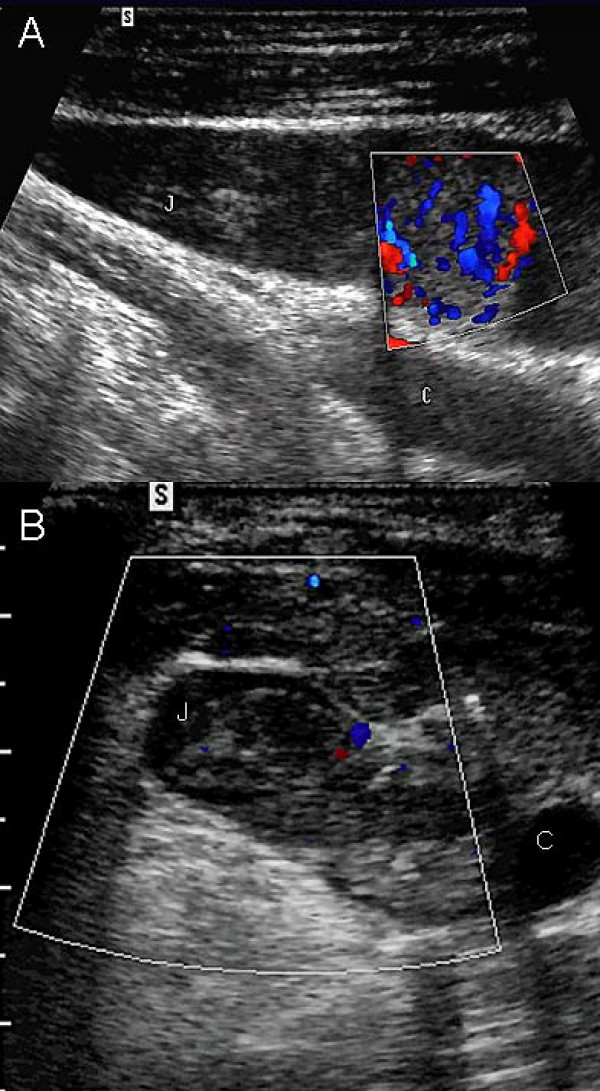
**Case 8 – US scan of early IJV tumor involvement**. **(A)** Longitudinal US scan of the IJV: Note the venous (blue) and arterial (red) vascularization of a tiny tumor thrombus into the IJV lumen. C: Carotid artery; J: Internal Jugular vein. **(B) **Axial horizontal US scan of the right jugulo-carotid vessels: Note the tumor thrombus invading laterally the IJV via the middle thyroid vein. C: Carotid artery; J: Internal Jugular vein.

Nine patients had thyroid malignancy (0.8%) diagnosed with cervical/mediastinal venous involvement (Table 1). Median age was 70 years (range; 34–81 years), median tumor diameter was 45 mm (range: 23–87 mm) and radical surgery with clear margins was performed in case 6 only. Median survival was seven months and median progression-free survival was 6 months. Histological types included papillary (n = 3), follicular (n = 3), anaplastic primary thyroid carcinomas (n = 1), and clear cell renal metastases to the thyroid (n = 2). Thyroid tumor staging was performed according to the TNM classification (Table 2). There were five cases (1, 3, 4, 5, 7) of poorly differentiated primary thyroid carcinomas (Table 1). All patients but two (cases 3, 6) had distant diffuse poorly differentiated metastases at the time vein involvement was diagnosed. Three patients (cases 1–3) presented with inaugural clinically typical superior vena cava syndrome and had large lateralized cervical tumors (mean diameter 81 mm)(Fig 1, 2). Three other patients (cases 4, 5, 7) presented with unilateral arm swelling due to venous thrombus extension down to the ipsilateral inominate vein. Three asymptomatic patients (cases 6, 8, 9) had vein invasion diagnosed by systematic Doppler-US. Multimodal therapy was performed in patients 3 and 6, including surgical excision thrombectomy followed by ^131^I, and additional external radiation beam therapy (EBRT) in patient 3. The latter developed diffuse metastases and died of disease progression at 50 months. Patient 6 was the sole patient aged less than 45 year-old who is still alive without disease at a 72 month follow-up. Palliative supportive care was performed for patient 2 owing to the advanced clinical and radiological presentation (Fig 1). Patients 4, 5, 7 and 8 had tracheal invasion, which was considered a contraindication to surgery at the time these patients were diagnosed with thyroid cancer. Patients 8 and 9 presented with malignant tumors mimicking primary thyroid carcinoma. Chemotherapy was combined with EBRT in those asymptomatic patients presenting with metastatic clear cell renal carcinoma. Metastases involved the bone, lungs and the thyroid gland. US-real-time guided fine needle aspiration provided the correct diagnosis in all cases but one (Fig 1B). Doppler US examination was performed in all patients. US revealed cervical venous obstruction, upper mediastinal venous obstruction in SVCS patients. US also helped to differentiate between venous compression and invasion and to define carotid artery encasement (Fig 2). In the neck, thyroid metastasis-as for patient 6-invaded the ipsilateral internal jugular vein (IJV) via the medial/superior thyroid vein lumen (Fig 3). Cases 1–5, 7 had extra-capsular tumor invading the soft tissues and the IJV wall on US. A 23 mm para-tracheal relapse follicular thyroid carcinoma mass invading the ipsilateral external jugular vein wall as well as distant metastases were found in patient 5.

**Table 1:** Cases of great vein involvement by thyroid malignancy at the Antoine Lacassagne Institute since 1991

(Case) TNM stage	Gender Age (years)	Status at Diagnosis	Histology Lobar tumor Size (mm)	Vein Involvement (Imaging)	Treatment Modality	Survival (months)	Progression-Free Survival (months)
(1) T4bN1bM1	M 68	Inaugural SVCS	Papillary (Insular) 87 mm	IJV, SCV, BCV (CT, US)	EBRT	4 DOD	0
(2) T4bN1bM1	M 71	Inaugural SVCS	Anaplastic 79 mm	IJV, BCV, SVC, RA (CT, US)	Supportive care	0.1 DOD	0
(3) T4aN1bM0	F 81	Inaugural SVCS	Follicular (P.D.) 76 mm	IJV, BCV, SVC (CT, US)	Thrombectomy + EBRT + ^131^I	50 DOD	40
(4) T4aN0M1	F 72	Arm Swelling LR/Mets	Follicular (P.D.) 23 mm	IJV (US)	EBRT	3 DOD	2
(5) T4aN0M1	M 70	Arm Swelling LR/Mets	Follicular (P.D.) 25 mm	EJV (US)	EBRT	47 DOD	6
(6) T2N1bM0	M 34	Asymptomatic	Papillary 28 mm	IJV (US)	S * + IJV resection + ^131^I	72 AWOD	72
(7) T4aN1bM1	F 75	Arm § neck swelling	Papillary (P.D.) 80 mm	IJV (US)	EBRT	1 DOD	0.5
(8) CCRC	M 63	Asymptomatic Mets	Mets 40 mm	IJV (US)	EBRT + Chemotherapy	18 DOD	4
(9) CCRC	M 69	Asymptomatic Mets	Mets 45 mm	IJV (US)	EBRT + Chemotherapy	7 DOD	2

Patient 4 had concomitant rectal cancer; Patient 5 presented with concomitant rectal and renal cancers. Both presented with rising serum levels of thyroglobulin from low levels to 4555 and 5500 u. Immuno-staining was positive for thyroglobulin on biopsy samples. *Patient 6 underwent total thyroidectomy, bilateral central and lateral neck dissection. Patients 8, 9 had previous CCRC and biopsy-proven metastases to the thyroid 46 and 84 months respectively after diagnosis of the primary. Poorly differentiated (PD) carcinomas of the thyroid (cases 1, 3, 4, 5 and 7) represent a heterogeneous but distinct group of tumors, clinically and histopathogenetically intermediate between follicular-derived well-differentiated and anaplastic carcinomas.

Mets: presence of diffuse distant metastases; S: Surgery; SVCS: superior vena cava syndrome; LR: Local recurrence. CCRC: Clear Cell Renal Carcinoma; DOD: dead of disease; AWOD: Alive without disease; EBRT: External beam radiation therapy

**Table 2:** TNM staging for thyroid cancer

**T: Primary tumor** All categories must be divided: (A) solitary, (b) multifocal tumor (the largest determines the classification).	**TX**: Primary tumor cannot be assessed **T0**: No evidence of primary tumor **T1**: Tumor ≤ 2 cm (greatest dimension) **T2**: 2 cm < Tumor ≤ 4 cm (greatest dimension, limited to thyroid) **T3**: Tumor > 4 cm or minimal extrathyroid invasion (eg. Extension to extrathyroid muscle and perithyroid soft tissue) **T4a**: Macroscopic invasion of adipose tissue, larynx, trachea, esophagus or recurrent laryngeal nerve **T4b**: Macroscopic invasion of prevertebral fascia, carotids, or mediastinal vessels *All anaplastic carcinomas are considered T4 tumors* *T4a: Intrathyroid anaplastic carcinoma-surgically resectable* *T4b: Extrathyroidal anaplastic carcinoma-surgically unresectable*	
**N: Regional Lymph Nodes** Regional lymph nodes are the central compartment, lateral cervical, and upper mediastinal lymph nodes	**Nx**: Lymph node status is unknown **N0**: No lymph node invasion **N1a**: Metastasis to level VI (recurrent nerve, pretracheal, paratracheal, prelaryngeal lymph nodes) **N1b**: Other lymphatic invasion (lateral-cervical and/or mediastinal)	
**M: Distant Metastasis**	**Mx**: Metastasic status is unknown **M0**: No metastasis **M1**: Distant Metastasis	
**Stage Grouping:**	Separate stage groupings are recommended for papillary or follicular, medullary, and anaplastic (undifferentiated) carcinoma.	
	**Papillary or Follicular Carcinomas**	
	**< 45 years**	**≥ 45 years**
**Stage I**	Any T, any N, M0	T1, N0, M0
**Stage II**	Any T, any N, M1	T2, N0, M0
**Stage III**	Non Available	T3, N0, M0
		T1-3, N1a, M0
**Stage IVA**	Non Available	T1-3, N1b, M0-1
		T4a, N0-1a, M0
		T1-4a, N1b, M0
**Stage IVB**		T4b, Any N, M0
**Stage IVC**		Any T, Any N, M1
	**Medullary Carcinomas**	
**Stage I**		T1, N0, M0
**Stage II**		T2, N0, M0
**Stage III**		T3, N0, M0
		T1-3, N1a, M0
**Stage IVA**	**Stage IVB**	T4a, N0-1a, M0
	**Stage IVC**	T1-4a, N1b, M0 T4b, Any N, M0
		Any T, Any N, M1
	**Anaplastic Carcinomas**	
**Stage IVA**		T4a, Any N, M0
**Stage IVB**		T4 b, Any N, M0
**Stage IVC**		Any T, Any N, M1

2002 American Joint Committee on Cancer (AJCC), Chicago Illinois-6^th ^Edition-Published by Springer-Verlag New York – )

The number stages of thyroid cancers depend on the type of thyroid cancer. There are different systems for papillary/follicular thyroid cancer, medullary and anaplastic thyroid cancer.

## Discussion

Venous involvement of thyroid tumors is rare and can be assessed by conventional cross-sectional imaging techniques namely magnetic resonance imaging (MRI) and multi-detector computed tomography (MDCT). Sagittal, coronal and 3D reconstructions along the long axis of the jugular and cava veins may be helpful to define the location, extent and nature (compression or invasion) of SVCS in cervical tumors. Nevertheless, a small thrombus may be missed with contrast-enhanced CT due to partial volume effect. Furthermore, metallic clips or patient swallowing artifacts may lead to misdiagnosis on MRI. High-frequency Doppler US is highly sensitive for thrombus detection in the neck veins since the vein has clear acoustic windows. Thus, at our institution, US has long been the imaging modality of choice for the diagnosis and follow-up of malignant thyroid nodules [2,3]. Doppler US was performed with Valsalva's maneuver in Trendelenburg's position. Such maneuvers increase the jugular vein's (and tributaries') diameter to differentiate between venous invasion and a strong tumor compression of the cervical vein. Even a small echogenic thrombus can be seen in the venous lumen and it can originate from either efferent thyroid veins such as in cases 6, 8, 9 or from extra-capsular tumor/malignant node spread (Fig 3). Cervical US showed venous thrombus in three asymptomatic patients (cases 6, 8, 9), revealed venous extension in three patients presenting with arm/neck swelling (cases 4, 5, 7) and confirmed superior vena cava syndrome in the remaining three patients (cases 1–3). Not only can cervical Doppler US show vein thrombosis but also its tumoral nature in showing a vascular arterialized invasive thrombus (Fig 3) [4]. Contrary to Hyer et al's assertion, US is an effective screening technique at initial diagnosis work-up and follow-up of thyroid malignancy for the diagnosis of SVC (and tributaries) obstruction despite the presence of nearby osseous structures and lung parenchyma [1]. Firstly, combined diminished respiratory phasicity and cardiac pulsatility of subclavian and jugular vein Doppler waveforms predict SVC obstruction with sensitivity, specificity, positive and negative predictive values of 75%, 100%, 91%, 100%, respectively [5]. Secondly, para-sternal Doppler US of internal thoracic veins is also sensitive to assess bloodstream within the SVC [6]. Thirdly, the use of an "endocavitary" US probe at the patient suprasternal notch, directed toward his upper mediastinum allows for clear depiction of the brachiocephalic veins, SVC flow and mediastinal compression (Fig 2A, B).

According to our experience, patients may present with various symptoms ranging from no symptoms, ipsilateral arm/neck swelling to typical SVC syndrome (one third each in our experience). Gross venous invasion is probably underdiagnosed in the routine practice: it has been reported in up to 1.5% of papillary cancers only [7]. Venous invasion is a poor prognosis factor in follicular neoplasms [8]. It is rather common in anaplastic carcinomas while even bulky cervical lymphoma never display jugular vein invasion [9]. Regarding the last two cases, thyroid masses were strongly hypervascular and invaded the ipsilateral IJV, thus mimicking a primary thyroid tumor. Noteworthy, clear cell renal primary tumors exhibit a venous tropism, leading to inferior vena cava thrombus extension. Thyroid metastases of renal origin behave like primary thyroid tumors and show a propensity to invade the internal jugular vein via the middle and superior thyroid veins (Fig 3B) [10]. Since the clinical presentation may be vague or misleading, we therefore highly recommend early neck US to prevent potential lethal complications such as pulmonary embolism or intracranial/intracardiac propagation of the thrombus (Figure 1A). We think that the low median survival in our study was mostly due to advanced stage diseases including aggressive primary thyroid malignancies contraindicated for surgery. Contraindication to surgical excision at our institution included thyroid cancer staged T4b (cases 1, 2), local recurrence and concomitant metastases, and diffuse metastases from renal cancer. Tracheal invasion was also considered a surgical contraindication at the time these patients were diagnosed with thyroid cancer, especially also as these patients were poorly-differentiated and/or metastatic cancers.

## Conclusion

Doppler US is a useful tool for the diagnosis of cervical venous invasion and extension to the central veins at initial work-up and during follow-up of thyroid malignancies, namely in asymptomatic patients or in patients presenting with arm and/or neck swelling (2/3 of patients). According to Hyer's study and our personal results and despite the small size of the series, it raises the question of whether more aggressive treatment modalities including surgery should be recommended in association with EBRT and radioiodine to prolong survival. Such question may be optimally answered with data from a large national registry.

For patients with a history of renal cell carcinoma, thyroid metastases should be ruled out.

## Abbreviations

MRI: Magnetic Resonance Imaging; MDCT: MultiDetector Computed Tomography; US: High Frequency Doppler Ultrasonography; EBRT: External Beam Radiation Therapy; ^131^I: RadioIodine Therapy; IJV: Internal Jugular Vein; EJV: External Jugular Vein; BCV: BrachioCephalic Vein; SCV: SubClavian Vein; SVC: Superior Vena Cava Syndrome; SVCS: SVC Syndrome; RA: Right Atrium; CCRC: Clear Cell renal Carcinoma-Mets Distant diffuse metastases; DOD: Dead Of Disease; AWOD: Alive Without Disease

## Competing interests

The authors declare that they have no competing interests.

## Authors' contributions

PYRM was involved in the original concept, initial and final draft and literature review, images and interpretation. JT, AB, GP, DB and OD prepared final draft. All authors read and approved the final manuscript.
